# Unraveling historical genetic divergence and gene flow patterns between island (Taiwan) and mainland (China) of *Fagus hayatae*

**DOI:** 10.3389/fpls.2025.1628728

**Published:** 2025-07-29

**Authors:** Rizal M. Suhardi, Li-Ping Ju, Tsai-Wen Hsu, Tze-Ying Chen, Huei-Chuan Shih, Zhi-Yong Zhang, Ya-Zhu Ko, Meng-Shin Shiao, Yu-Chung Chiang

**Affiliations:** ^1^ Department of Biological Sciences, National Sun Yat-sen University, Kaohsiung, Taiwan; ^2^ Forest Protection Division, Taiwan Forestry Research Institute, Taipei, Taiwan; ^3^ Taiwan Biodiversity Research Institute, Nantou, Taiwan; ^4^ Department of Natural Resources, National Ilan University, Yilan, Taiwan; ^5^ Department of Nursing, Meiho University, Pingtung, Taiwan; ^6^ Key Laboratory of Ecology of Rare and Endangered Species and Environmental Protection (Guangxi Normal University), Ministry of Education, Guilin, China; ^7^ University Engineering Research Center of Bioinformation and Genetic Improvement of Specialty Crops, Guangxi, Guilin, China; ^8^ Research Laboratory Section, Offices of Health Science Research, Faculty of Medicine, Ramathibodi Hospital, Mahidol University, Bangkok, Thailand; ^9^ Department of Biomedical Science and Environment Biology, Kaohsiung Medical University, Kaohsiung, Taiwan

**Keywords:** *Fagus hayatae*, phylogeography, genetic diversity, population structure, gene flow, relict species

## Abstract

The disjunctive distribution of *Fagus hayatae* between Taiwan and mainland China provides a unique framework for understanding phylogeographic patterns and evolutionary processes in relict tree species. This study investigated the genetic diversity, population structure, and phylogeographic history of *F. hayatae* using microsatellite and chloroplast DNA markers. Analysis of 249 samples from nine populations revealed that Taiwanese populations possess higher genetic diversity with significant inbreeding, while mainland Chinese populations showed lower diversity and inbreeding levels. Chloroplast DNA analysis identified eight haplotypes, with unique variants in Taiwanese populations. AMOVA confirmed significant genetic differentiation between the two regions, revealing four distinct clustering patterns and three clear phylogenetic clades, including an early-diverging Taiwanese TS population. Molecular divergence time estimation based on chloroplast DNA sequences indicates a temporal divergence pattern within *F. hayatae* populations, with the Taiwanese TS lineage exhibiting an earlier separation event, followed by subsequent divergence between other Taiwanese populations (PCTS and AW) and mainland Chinese populations (CH). These phylogeographic events temporally correspond with significant paleoclimatic and geotectonic episodes in East Asia. Demographic and historical biogeography inference revealed a recent population decline in Taiwan, in contrast to more stable population dynamics on the mainland, while historical gene flow between the regions remains limited, and suggested several dispersals and vicariant events influencing the current genetic structure. These findings not only delineate the genetic structure shaped by historical geographic isolation and contemporary evolutionary processes but also emphasize Taiwan’s role as a genetic refugium for *F. hayatae*.

## 1 Introduction

*Fagus hayatae* Palib. ex Hayata is a large deciduous tree species recognized as a relict taxon, with a disjunctive distribution spanning Taiwan and several provinces in China, including Hubei, Sichuan, Gansu, Shaanxi, and Zhejiang (Liao, 1996; Zhang, 2017). As the only *Fagus* species exhibiting a discontinuous distribution pattern extending from the subtropical mountainous regions of China to Taiwan, it serves as a crucial model for investigating phytogeographic connections between mainland China and island Taiwan, as well as understanding vegetation succession patterns (Shen et al., 2015).

The species predominantly occurs in mixed evergreen-deciduous broadleaf forests and montane deciduous broadleaf forests, where it serves as a keystone species in these forest communities (Zhang, 2001). The species faces significant conservation challenges due to habitat fragmentation caused by anthropogenic disturbances and global climate change, which severely impacts its natural regeneration processes. In response to these conservation challenges, it has been designated as a Class II protected species in the Chinese List of Wild Plants and has been assessed as Vulnerable by the IUCN (Liao, 1996). In Taiwan, the species is a rare and valuable endemic with a very limited distribution, found only in small, isolated patches on ridgetops at altitudes of 1,340 to 2,000 meters in the northern and northeastern regions (Liao, 1996). The species also faces reproductive difficulties, including low fruiting rates and poor seed germination, which significantly hinder conservation efforts and natural population recovery (Chen et al., 2011). Among the geographically isolated populations in Chahtianshan (Taoyuan), Tungshan, Tahpaishan, Lankanshan (Yilan), Niautzueishan (Hsinchu), and Ayushan, only the Chahtianshan population is currently under formal protection within the Chahtianshan Nature Reserve (Liao, 1996).

Current studies on *Fagus* in China have primarily addressed aspects such as community structure (Ke and Werger, 1999; Shen et al., 2015), species composition (Hukusima et al., 2005; Fang and Lechowicz, 2006), and population dynamics, along with regeneration strategies and coexistence with other tree species (Shen and Jin, 1995; Jinsheng et al., 1998; Shen and Fang, 2001; Yu et al., 2009; Ying et al., 2016). While certain investigations have examined the distribution, morphology, ecology and genetic diversity of *F. hayatae* (Kato et al., 2000; Denk, 2003; Jiang et al., 2014; Ying et al., 2016; Li et al., 2023), critical gaps remain regarding its phylogenetic relationships, genetic structuring, and evolutionary history, especially in the context of the geographic divide between mainland China and island Taiwan. These unresolved aspects highlight the need for comprehensive genetic analyses to clarify population differentiation, gene flow, and the evolutionary trajectory of *F. hayatae*, which are essential for understanding the biogeographical links and informing conservation strategies for this relict species.

Due to the highly fragmented distribution and morphological variation of *F. hayatae* between island Taiwan and mainland China, its taxonomic classification remains disputed. Some researches considered the populations to be a distinct species while others suggest they should be classified as a subspecies (Shen, 1992). This complexity is further underscored by recent taxonomic revisions within the group. Li et al. (2023) used molecular and morphological evidence to demonstrate that the rare mainland taxon *F. chienii* is conspecific with *F. hayatae*. Such findings highlight the need to clarify the status of the deeply divergent island populations relative to their mainland counterparts. Moreover, significant differences in ecological habits and community composition have been observed. Taiwanese populations are typically found in mixed evergreen-deciduous forests with species such as *Quercus*, *Lithocarpus*, and *Castanopsis*, in warmer, more humid environments with less seasonal variation in precipitation and temperature. In contrast, mainland Chinese populations often occur with other Fagus species and in deciduous or evergreen oak forests, adapting to colder and drier climates (Shen et al., 2015). The ecological and habitat differentiations imply that the two regional populations may have experienced divergent evolutionary trajectories. Yet, the current genetic data are insufficient to fully resolve the genetic structure and historical evolution between the Taiwanese and mainland populations of *F. hayatae*. Furthermore, as habitat fragmentation and climate change intensify, employing comprehensive genetic studies to clarify the inter-population relationships, gene flow direction, and evolutionary dynamics is critical for the conservation and management of *F. hayatae*.

This study proposes three primary hypotheses: First, *F. hayatae* populations in Taiwan and mainland China have undergone significant genetic differentiation due to the combined effects of geographical barriers and historical climate change. Second, the degree of gene flow within *F. hayatae* populations in Taiwan and mainland China is affected by habitat fragmentation, population size reduction, and ecological adaptation, forming diverse genetic patterns at fine scales. Third, phylogeographic reconstruction will reveal the pathways and differentiation processes, illuminating the evolutionary relationships between insular and continental populations. To address these hypotheses, we will employ an integrated analytical approach using microsatellite and chloroplast markers with three main objectives: (1) evaluate the genetic diversity of *F. hayatae* populations in mainland China and Taiwan to understand the level of genetic variation within each population, (2) investigate the degree of genetic differentiation between mainland China and Taiwan *F. hayatae* populations and their gene flow patterns, clarify whether gene flow is limited by geographical isolation, and assess its impact on genetic differentiation; and (3) reconstruct the phylogeographic history of *F. hayatae*, inferring its dispersal and differentiation history and exploring the evolutionary relationships between Taiwanese and mainland populations. Through these multi-level analyses, the study will provide critical scientific evidence for the genetic conservation strategies and evolutionary history of *F. hayatae*.

## 2 Materials and methods

### 2.1 Population sampling and DNA extraction

Leaf samples of *F. hayatae* were collected from three native habitats in Taiwan and six in mainland China (**Table 1**). In Taiwan (TW region), sampling was conducted in three populations separated by the Lanyang River: 101 individuals from the Tongshan (TS) population (south of the river), 16 from the Ayushan Mountain West Peak (AW) population (north of the river), and 40 from the Peichatienshan (PCTS) population (north of the river). In mainland China, samples were collected from two regions: the Zhejiang Province (ZJ region), with 8 individuals from Sihaishan (CH), 23 from Qingliangfeng (DB), and 3 from Tianmu Mountain (TM); and the Hubei and Sichuan Provinces (HS region), with 16 individuals from Shennongjia (SNJ), 18 from Big-small Langou Nature Reserve (LG), and 37 from Micangshan (MC). Two samples each of *F. multinervis* (UL) and *F. sylvatica* (UK) served as outgroups. A total of 249 samples were used in the analysis (**Table 1**). Fresh young leaves were collected, rapidly dehydrated using silica gel, and stored for subsequent genomic DNA extraction.

**Table 1:** Sampling information and haplotypes of *Fagus hayatae* and outgroup of *F. multinervis* Nakai (UL) and *F. sylvatica* (UK) for cpDNA and nuclear microsatellite (nSSR) analyses.

Population code	Population	Latitude(N)/longitude(E)	Sample size (n) of nSSR/cpDNA	cpDNA haplotypes (no. of individuals)
Taiwan (TW region)				
TS	Tungshan, Taiwan	24°30’/120°39’	37/101	Hap3(101)
PCTS	Peichatienshan, Taiwan	24°47’/121°27’	46/40	Hap7*(40)
AW	Ayushan, Taiwan	24°47’/121°36’	16/16	Hap7*(16)
China (ZJ region)				
CH	Sihaishan, Wenzhou, Zhejiang Province, China	28°32’/120°44’	8/8	Hap4(8)
DB	Qingliangfeng nature reserve, Hangzhou, Zhejiang Province, China	30°05’/118°52’	23/23	Hap5(23)
TM	Tianmu Mountain, Hangzhou, Zhejiang Province, China	30°20’/119°24’	3/3	Hap6*(3)
China (HS region)				
SNJ	Shennongjia Forestry District, Hubei Province, China	31°44’/110°40’	13/16	Hap6*(6), Hap8(10)
LG	Big-small Langou Nature Reserve, Nanjiang, Sichuan Province, China	32°42’/106°51’	18/18	Hap6*(1)
MC	Micangshan, Nanjiang, Sichuan Province, China	32°42’/106°59’	20/37	Hap6*(37)
Outgroup				
UK	Royal Botanic Gardens, Kew, England	51°28’/0°18’	-/2	Hap1(2)
UL	Ulleungdo, Korean	37°30’/130°52’	-/2	Hap2(2)
**Overall**			184/249	Hap1- Hap8 (249)

The shared haplotypes are indicated in bold and marked *. - = no individuals to be used for nuclear microsatellite analysis.

Genomic DNA was extracted using a modified CTAB method (Doyle and Doyle, 1987), dissolved in 200 μl of Tris-EDTA (TE) buffer, and stored at -20°C. DNA quality was assessed via 1% agarose gel electrophoresis using a Lambda marker (200 μg/mL, Promega) and diluted to 10 ng/μl in TE buffer for subsequent experiments.

### 2.2 Microsatellite genotyping and chloroplast DNA fragments sequencing

To assess the levels of genetic variability and population structure in *F. hayatae*, genotyping was performed using ten previously characterized polymorphic microsatellite markers described by Ju et al. (2012) (Ju et al., 2012). Although 249 individuals were sampled for this study, DNA of sufficient quality and quantity for the sensitive microsatellite analysis could only be obtained from 184 samples, as detailed in **Table 1**. These 184 samples (**Table 1**) underwent PCR amplification using a Labnet MultiGene 96-well Gradient Thermal Cycler with the following parameters: initial denaturation at 94°C for 2 min, followed by 35–40 cycles of 94°C for 45s, 54-64°C for 1 min, and 72°C for 30s, concluding with a final extension at 72°C for 7 min. Microsatellite genotyping followed the methodology outlined by Mantiquilla et al. (2022) (Mantiquilla et al., 2022).

To evaluate the phylogeographic structure and dynamics of *F. hayatae* populations in Taiwan and mainland China, the *atp*B-*rbc*L regions of the plastid genome were amplified using the forward primer 5’- ACCGGACCAATGATTTGAGCC-3’ and the reverse primer 5’-TACAGTTGTCCATGTACCAG-3’. A PCR reaction mixture totaling 50 µL comprised 1 µL of template DNA, 5 µL of 10× reaction buffer, 5 µL of dNTP mix (2 mM), 5 µL of each forward and reverse primer (2 mM), 1 µL of *Taq* polymerase (0.2 U/µL; Promega), and 28 µL of sterile water. The PCR amplification protocol included an initial denaturation at 95°C for 2 min, followed by 38 cycles of denaturation at 94°C for 45 s, annealing at 54°C for 1 min, and extension at 72°C for 1 min, culminating in a final extension at 72°C for 7 min. All PCR products were purified using the HiYield™ Gel/PCR DNA Fragments Extraction Kit (RBC Bioscience). Both strands of the purified PCR fragments were sequenced using the ABI PRISM 3730XL DNA sequencer (Applied Biosystems, Foster City, CA, USA).

The resulting forward and reverse sequence data were assembled into contigs using SeqMan software from the DNASTAR package (DNASTAR Inc., Madison, WI, USA). Multiple sequences were aligned and retrieved from the GenBank database utilizing MEGA 11 (Kumar et al., 2018) for subsequent phylogenetic analyses. The optimal nucleotide substitution model was determined using jModelTest v2.1.7 (Darriba et al., 2012) based on the Bayesian Information Criterion (BIC), identifying the HKY + G model, which was subsequently applied to each Beast analysis conducted with BEAST v1.10.4 (Suchard et al., 2018). All sequences of *atp*B-*rbc*L regions submitted to GenBank (GenBank accession numbers: LM992847-LM992854).

### 2.3 Data analyses

#### 2.3.1 Genetic diversity and nucleotide diversity

Microsatellite analysis was conducted using GenAlEx v6.5 (Smouse et al., 2017) to evaluate key genetic parameters at the population level, including observed heterozygosity (*H_O_*), expected heterozygosity (*H_E_*), number of alleles (*A*), and effective number of alleles (*A_E_*). Additionally, we calculated the fixation index (*F_IS_*) to evaluate inbreeding levels within populations. Population-specific genetic characteristics were further assessed through allelic richness (*Ar*) and private allelic richness (*Ap*) calculations using HP-Rare (Kalinowski, 2005). Deviations from Hardy-Weinberg equilibrium, based on gene frequency across loci, were examined via a chi-square (*χ²*) test implemented in GenAlEx v6.5.

For chloroplast DNA data analysis, genetic diversity and polymorphism were assessed using DnaSP v6.12.03 (Rozas et al., 2017). Specifically, the diversity among populations of *F. hayatae* was evaluated by determining the number of haplotypes (*h*), the segregating sites (*S*), haplotype diversity (*Hd*), genetic diversity within populations (*H_S_*), total genetic diversity (*H_T_*), haplotype richness (*Hr*), private haplotype richness (*Hp*), and nucleotide diversity (*π*).

#### 2.3.2 Genetic differentiation and genetic groups analysis

To determine the distribution of genetic variation and population structure, we performed a hierarchical analysis of molecular variance (AMOVA) in Arlequin v3.5 (Excoffier and Lischer, 2010). This analysis quantified genetic variance components at different hierarchical levels, with statistical significance of *F* statistics evaluated through 999 permutation tests.

To evaluate geographic population structure, we conducted a spatial analysis of molecular variance (SAMOVA) utilizing SAMOVA v10 (Dupanloup et al., 2002). We configured the analysis to execute independent iterations with *K* values from 2 to 5, implementing 500 simulations per iteration. We employed squared size differences as our molecular distance measure, which is particularly well-suited for microsatellite data analysis. This approach enabled us to determine the optimal *F_CT_* value and identify significant patterns in genetic structure across populations.

Additionally, we employed Bayesian clustering analyses using Structure (Pritchard et al., 2000) to classify individuals into source populations based on allele frequencies. This approach utilizes Markov Chain Monte Carlo (MCMC) estimation to analyze the distribution of genetic variation and to group individuals with similar genetic characteristics. The algorithm assigns individuals to *K* ancestry clusters, estimates allele frequencies, and reassigns individuals under the assumptions of Hardy-Weinberg equilibrium and linkage equilibrium. In our study, we implemented an admixture model with population identifiers, performing analyses with 10^5^ burn-in periods and 10^6^ MCMC replicates. Structure Harvester was utilized to evaluate likelihood values across multiple *K* values and iterations (Earl and VonHoldt, 2012). To address potential multimodality issues, CLUMPP was employed to align multiple replicates of the selected *K* cluster (Jakobsson and Rosenberg, 2007). For visual representation of the Structure output, bar plots were generated using the POPHELPER R package (Francis, 2017).

#### 2.3.3 Phylogenetic analysis and divergence time estimates

A minimum spanning network was constructed to analyze the haplotype relationships of *F. hayatae* using POPART v1.7 (Leigh et al., 2015). The analysis utilized pairwise differences between haplotypes to calculate mutational steps connecting different haplotype groups. The resulting network visualization was optimized in Adobe Illustrator 2025 to ensure optimal presentation of the evolutionary relationships.

The haplotype phylogeny based on the chloroplast DNA dataset was analyzed using Bayesian methods in MrBayes v3.2.7a (Ronquist et al., 2012). The analysis consisted of two separate runs with four chains each, running for 40 million generations. Tree structures were recorded every 100,000 generations. For quality assurance, we excluded the first 25% of generations from each run. We confirmed the analysis quality by monitoring two key metrics: the average standard deviation of split frequencies (ASDSF), which needed to be under 0.01, and the potential scale reduction factor (PSRF), which needed to stay near 1.0 (Drummond and Rambaut, 2007). Tree topology data, including branch rates, node heights, and associated statistical metrics, were visualized and analyzed using FigTree v1.4.4 (Rambaut, 2018).

BEAST v1.10.4 (Suchard et al., 2018) was employed to estimate divergence times among *F. hayatae* haplotypes. For the non-coding chloroplast DNA regions, we utilized a substitution rate of 1.52 × 10–^9^ substitutions per site per year (with bounds of 1.46-1.58 × 10–^9^ s/s/y) based on Yamane et al. (2006) (Yamane et al., 2006), as taxon-specific rates were unavailable. The analysis involved three independent MCMC runs of ten million generations, sampling genealogies every 1,000 generations, with a 10% burn-in period. Results were processed using Tracer v1.5 for analysis, LOGCOMBINER v1.6.1 (Drummond and Rambaut, 2007) for file integration, and TREEANNOTATOR v1.6.1 (Drummond and Rambaut, 2007) for tree summarization. The final phylogenetic visualization was generated in FigTree v1.4.4 (Rambaut, 2018), enabling comprehensive interpretation of the species’ evolutionary timeline.

#### 2.3.4 Demographic history analyses

Historical demographic events were investigated through two neutrality statistical tests implemented in DnaSP v6.12.03 software (Rozas et al., 2017): Tajima’s *D* test (Tajima, 1989) and Fu & Li’s D* test (Fu and Li, 1993). These analytical methods assess departures from neutrality, providing insights into population dynamics such as expansion events, population bottlenecks, or selective forces.

Further examination of demographic and spatial expansion hypotheses was performed using mismatch distribution analysis, employing 1,000 parametric bootstraps (Schneider and Excoffier, 1999). The goodness-of-fit for each expansion model was assessed using the sum of squared deviations (SSD) and Harpending’s raggedness index (*H*_Rag_) with Arlequin version 3.5 (Excoffier and Lischer, 2010). P-values were calculated as the proportion of simulations where the SSD exceeded the observed value. Additionally, *H*_Rag_ was used to evaluate the smoothness of the mismatch distributions, providing further insights into population dynamics (Harpending, 1994).

To infer historical demographic changes, a Bayesian skyline plot (BSP) was generated using BEAST v1.10.4 software. The HKY + G substitution model was selected, with an assumed nucleotide substitution rate of 1.52 × 10^-9^ substitutions per site per year (Yamane et al., 2006). Three independent Markov chains were run for 2.0 × 10^7^ generations, with samples collected every 1,000 generations; the first 10% of iterations were discarded as burn-in to ensure convergence.

A coalescent-based isolation-with-migration (IM) model was implemented using the IMa program (Hey and Nielsen, 2007) to estimate key demographic parameters, including effective population sizes and migration rates, between two *F. hayatae* populations: Taiwan (TW region; population 1) and mainland China (ZJ and HS regions; population 2). The Stepwise Mutation Model (Kimura and Ohta, 1978) was applied, with the effective population size parameter (*θ*) assigned to both present and ancestral populations and migration rate parameters (*m*) defined between source and target populations. Markov Chain Monte Carlo (MCMC) runs included a burn-in period of 2 million iterations, followed by an additional 20 million iterations for parameter estimation. All parameters were scaled using a mutation rate of 1.52 × 10^-9^ substitutions per year for chloroplast DNA (Yamane et al., 2006) and 8.87 × 10^-4^ mutations per allele per generation for microsatellite loci (Marriage et al., 2009).

#### 2.3.5 Phylogeographical inference

To investigate the historical biogeographical patterns and mechanisms shaping the present-day distribution of *F. hayatae* populations, we implemented statistical dispersal-vicariance analysis (S-DIVA) through RASP software (Yu et al., 2015). The analysis delineated the species’ geographical ranges into five distinct areas: two outgroup regions comprising Korea and England (areas A and B, respectively), Taiwan (area C), Zhejiang Province in China (area D), and a combined region of Hubei and Sichuan provinces in China (area E). In the reconstruction process, a maximum of three areas per node was permitted to account for potential ancestral range overlaps. All other parameters in the analysis were optimized automatically by the software to best account for the data.

## 3 Results

### 3.1 Genetic diversity and nucleotide diversity

Microsatellite data revealed varying levels of genetic diversity among *F. hayatae* populations across three regions (Taiwan [TW], Zhejiang [ZJ], and Hubei/Sichuan [HS]) (**Table 2**). Hereafter, we refer to TW populations as “island populations” and Chinese populations (ZJ and HS) as “mainland populations”. The island populations showed greater genetic diversity, with higher numbers of different alleles (*A* = 12.37), number of effective alleles (*A_E_* = 6.55), allelic richness (*Ar* = 4.06), and private allelic richness (*A_P_* = 2.14) comparing to the mainland populations. Among the island populations, PCTS had the highest genetic diversity (*A* = 18.80, *A_E_* = 8.85, *Ar* = 4.57, *A_P_* = 2.23), whereas the AW population had lower genetic diversity (*A* = 5.40, *A_E_* = 3.70, *Ar* = 3.20, *A_P_* = 1.77). Among mainland populations, DB showed relatively higher genetic diversity (*A* = 5.90, *A_E_* = 3.27, *Ar* = 2.96, *Ap* = 0.66).

Hardy-Weinberg equilibrium (HWE) tests revealed significant deviations from equilibrium in all island populations, with observed heterozygosities (*H_O_*) consistently lower than expected heterozygosities (*H_E_*). Similarly, all mainland populations exhibited lower *H_O_* than *H_E_* and deviated significantly from HWE, except the TM population, which showed no significant deviation from HWE (**Table 2**). Analysis of fixation index (*F_IS_*) values revealed a significantly higher *F_IS_* value in island populations (0.96), with the AW population exhibiting the highest value (*F_IS_* = 0.98), suggesting a high degree of inbreeding in the island populations. While most mainland populations showed significant *F_IS_* values, the CH, TM, and SNJ populations exhibited low and non-significant values (**Table 2**). Taken together, *F. hayatae* island populations showed higher genetic diversity than mainland populations, along with significant levels of inbreeding.

**Figure 1:**
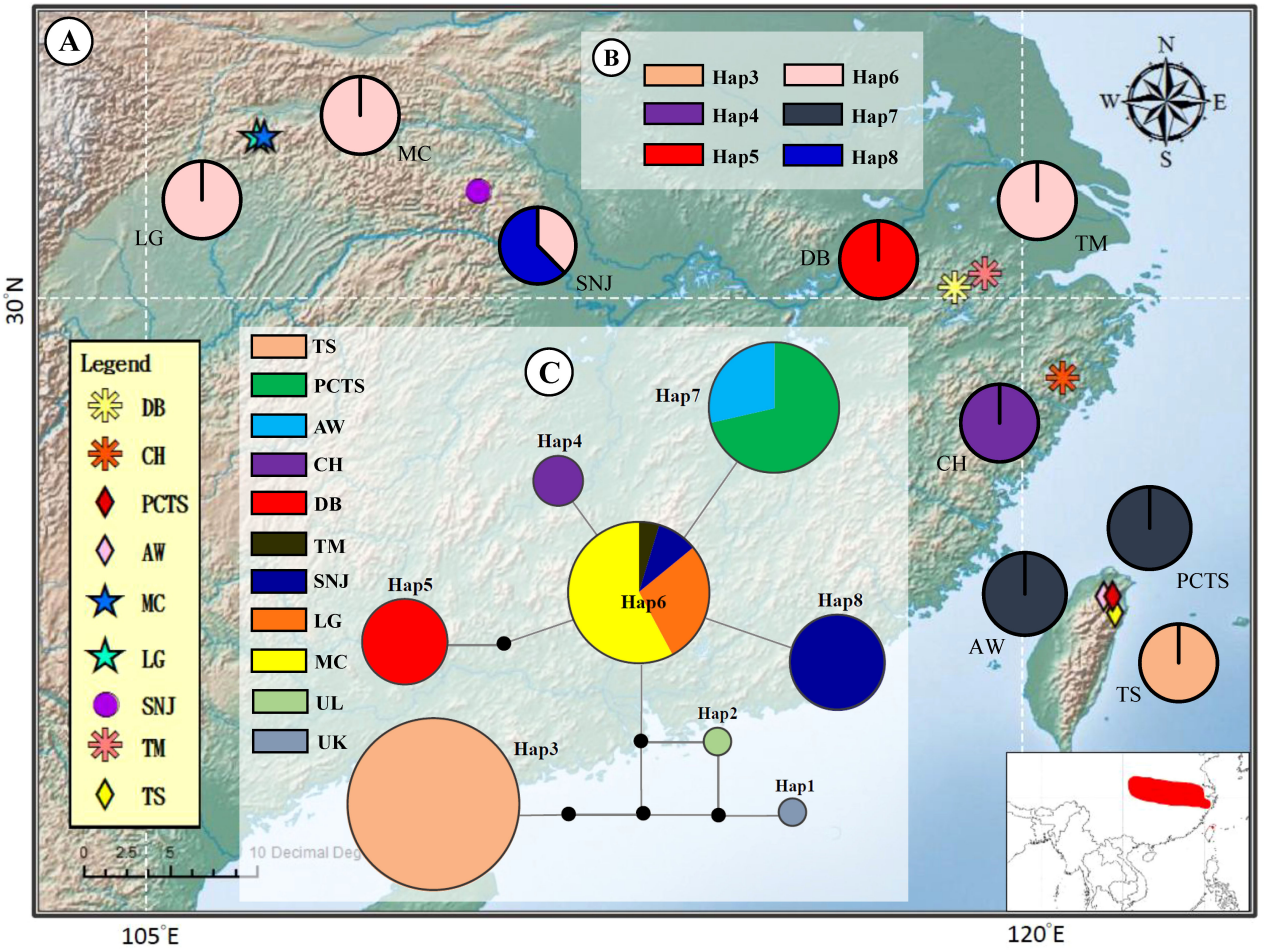
Geographic distribution and evolutionary relationships of chloroplast DNA haplotypes of *Fagus hayatae*. **(A)** Sample localities of the 9 populations. **(B)** Haplotype distribution of each populations. The different colors in pies charts represent haplotypes. **(C)** Unrooted 95% plausible TCS network. The size of the circles are proportional to the frequency of a haplotype over all the populations. The colors represent populations. The black dots are the missing or inferred haplotypes and each line represents one mutational step. Population names correspond to **Table 1**.

Examination of chloroplast DNA data across nine *F. hayatae* populations in island and mainland revealed six distinct haplotypes (**Table 1**, **Figure 1**). The analysis demonstrated that island populations harbored exclusive haplotypes (Hap3 and Hap7), distinguishing them from mainland populations. Notably, island-specific haplotype Hap3 was found solely in the TS population, absent from other island populations (PCTS and AW), underscoring the unique genetic composition of the TS population. Four haplotypes were identified in mainland populations, with Hap6 demonstrating widespread distribution across ZJ and HS regions, while CH, DB, and SNJ populations possessed unique haplotypes (Hap4, Hap5 and Hap8) (**Table 1**, **Figure 1**).

Further genetic analysis revealed that the island populations displayed the highest genetic diversity (*H_T_*, *Hr*, *Hp*, *Hd* = 0.84, 1.55, 1.55, 0.46) and nucleotide diversity (*π* = 2.48 × 10^-3^, *θw* = 0.95 × 10^-3^), while the HS region showed the lowest (*π* = 0.26 × 10^-3^, *θw* = 0.22 × 10^-3^) based on chloroplast DNA data (**Supplementary Table S1**). The observed reduced overall nucleotide diversity of *F. hayatae* populations indicates potential historical genetic bottlenecks or recent population isolation events. Furthermore, the high *G_ST_* and *N_ST_* values (>0.5) detected in all populations (**Supplementary Table S1**) indicate significant genetic differentiation and fragmentation among these populations.

**Table 2:** Genetic diversity parameters estimated from microsatellite data of *F. hayatae*.

Region/Population	*A*	*A_E_*	*Ar*	*Ap*	*H_O_*	*H_E_*	*F_IS_*
**TW region**	12.37	6.55	4.06	2.14	0.07	0.78	0.96*
TS	12.90	7.11	4.41	2.43	0.09	0.85^(10*)^	0.95*
PCTS	18.80	8.85	4.57	2.23	0.11	0.86^(10*)^	0.96*
AW	5.40	3.70	3.20	1.77	0.01	0.64^(9*)^	0.98*
**ZJ region**	3.33	2.19	2.23	0.47	0.17	0.38	0.36*
CH	2.10	1.45	1.73	0.32	0.14	0.26^(6*)^	0.08*
DB	5.90	3.27	2.96	0.66	0.15	0.58^(9*)^	0.70*
TM	2.00	1.84	2.00	0.42	0.23	0.30	0.30*
**HS region**	4.20	2.54	2.39	0.35	0.21	0.42	0.42*
SNJ	3.30	2.11	2.19	0.24	0.23	0.38^(5*)^	0.22*
LG	4.70	2.96	2.60	0.53	0.22	0.48^(8*)^	0.51*
MC	4.60	2.55	2.37	0.27	0.17	0.40^(6*)^	0.52*

*A*, number of different alleles; *A_E_*, number of effective alleles; *Ar*, allelic richness; *Ap*, private allelic richness; *Ho*, observed heterozygosity; *H_E_*, expected heterozygosity; *F_IS_*, fixation index, * *p*<0.05. Numbers in parentheses after *H_E_* values indicate the number of microsatellite loci that significantly deviated from Hardy-Weinberg equilibrium out of 10 loci tested.

### 3.2 Genetic differentiation and genetic structure pattern

Hierarchical AMOVA was performed using both chloroplast DNA and microsatellite data to assess genetic differentiation across various hierarchical levels defined by geographical regions and genetic groups (**Supplementary Table S2**).

Using chloroplast DNA sequences, analyses based on different spatial grouping models—*K*_geo_ = 2 (Taiwan & China), *K*_geo_ = 3 (TW, HS, and ZJ regions), and *K*_geo_ = 9 (all nine populations)—revealed that the majority of genetic variation was accumulated among populations within groups (75.97%, 81.95%, and 99.13% for the *K*_geo_ = 2, *K*_geo_ = 3, and *K*_geo_ = 9 patterns, respectively), with both among and within population variability being high and statistically significant (Φ*_SC_* and Φ*_ST_* > 0.99). In contrast, analysis based on genetic groupings (*K*_S_ = 2, as determined by STRUCTURE analysis; TS and PCTS in one cluster, and AW, CH, DB, TM, SNJ, MC, and LG in the other) indicated that most of the genetic variation (71.31%) accumulated among groups, with consistently high and significant variability at all hierarchical levels (Φ*_CT_* = 0.71, Φ*_SC_* = 0.98, and Φ*_ST_* = 0.99) (**Supplementary Table S2**).

Interestingly, microsatellite polymorphisms revealed a distinctive pattern of genetic variation (**Supplementary Table S2**). Regardless of whether populations were grouped by geographical models (*K*_geo_ = 2, *K*_geo_ = 3, or *K*_geo_ = 9) or by genetic clusters (*K*_S_ = 3, determined by STRUCTURE analysis: TS and PCTS in one cluster, AW in another, and CH, DB, TM, SNJ, MC, LG in the third; *K*_SA_ = 4, determined by SAMOVA analysis: PCTS, AW, and TS each in separate clusters, with CH, DB, TM, SNJ, MC, LG in the fourth), the analyses consistently showed that the majority of genetic variation was accumulated within populations (69.04%, 69.78%, 73.62%, 72.17%, and 72.68% for *K*_geo_ = 2, *K*_geo_ = 3, *K*_geo_ = 9, *K*_S_ = 3, *K*_SA_ = 4 group patterns). Notably, significant variability was observed across groupings, among populations, and within populations (Φ*_CT_*, Φ*_SC_*, and Φ*_ST_* ranging from 0.06 to 0.30). These findings indicate that while chloroplast DNA primarily captures differences between populations, microsatellite markers are better at detecting finer-scale intra-population genetic variability (**Supplementary Table S2**).

**Figure 2:**
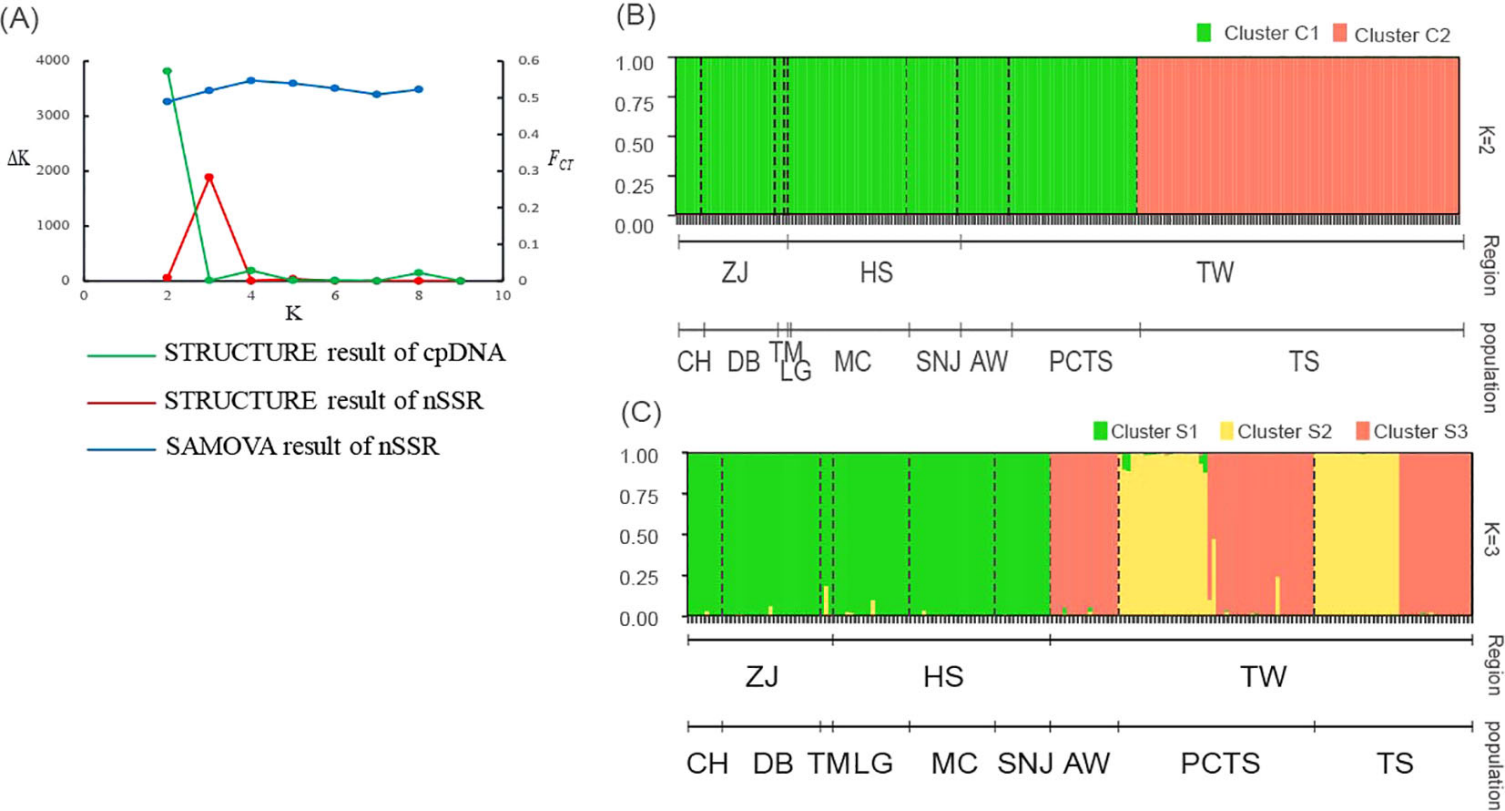
Population structure inference for *F hayatae* based on chloroplast DNA and microsatellite data. **(A)** Plots of *ΔK* and *F_CT_* values showing optimal *K* values inferred by STRUCTURE analysis for chloroplast DNA (*K*=2) and microsatellite data (*K*=3), and by SAMOVA analysis for microsatellite data (*K*=4). **(B)** STRUCTURE analysis results for chloroplast DNA data showing two genetic clusters (C1 and C2), where each individual is represented by a vertical line partitioned into colored segments indicating the assignment probability to each cluster. **(C)** STRUCTURE analysis results for microsatellite data revealing four genetic clusters (S1-S3). Black dashed lines indicate the nine sampled populations. The y-axis represents the probability of cluster membership, while the x-axis shows the geographic regions (ZJ, Zhejiang Province; HS, Hubei and Sichuan Provinces; TW, Taiwan) and their corresponding populations (CH, DB, TM, LG, MC, SNJ, AW, PCTS, TS).

After confirming significant genetic variation among populations, we investigated the population structure of *F. hayatae* using two analyses: SAMOVA and STRUCTURE. SAMOVA was used as a preliminary analysis with both microsatellite and chloroplast DNA data. Based on *F_CT_* as the criterion for optimal grouping, the chloroplast DNA data did not show a clear clustering pattern (**Supplementary Table S3**). In contrast, the microsatellite data revealed four distinct clusters (*K*=4), with the highest *F*_CT_ value of 0.55 (*p* < 0.05) (**Supplementary Table S3**; **Figure 2A**). Under this model, the three populations from the island (PCTS, AW, and TS) were clearly distinguished from each other, while all populations from the mainland (CH, DB, TM, SNJ, MC, LG) grouped together. This initial clustering analysis supports the classification of island and mainland populations into distinct genetic groups.

We also used STRUCTURE to further examine the genetic structure of the populations. The optimal number of clusters (*K*) was determined by the value of *ΔK*. For chloroplast DNA, the *ΔK* method identified K = 2 (*ΔK* = 3818.27), with the TS population from island forming a distinct cluster (Cluster C2) separate from the other groups (**Supplementary Table S3**; **Figures 2A, B**). In contrast, for the SSR data, the optimal number of clusters was *K* = 3 (*ΔK* = 1882.04). In the island populations (PCTS, AW, and TS), two distinct genetic clusters were observed (S2, and S3). AW was composed solely of cluster S3, while the PCTS and TS populations displayed more varied genetic components (cluster S2 and S3). All mainland populations were homogeneous, forming a single cluster (Cluster S1) (**Supplementary Table S3**; **Figures 2A, C**). These clustering analyses revealed a distinct population genetic structure with a clear separation between the island and mainland, consistent across both genetic markers.

### 3.3 Phylogenetic relationships and differentiation time estimates

The haplotype network of *F. hayatae* showed a distinct grouping of island populations (**Figure 1C**). These island populations possessed unique haplotypes, Hap3 and Hap7. Hap3, found only in the TS population and separated from others by more than three mutational steps, suggested long-term isolation or independent evolution. In contrast, Hap7 was closer to mainland haplotypes, suggesting a historical gene flow between island and mainland populations. In addition, high genetic differentiation was evident, with limited haplotype sharing among populations except for Hap6, which was common in the ZJ and HS regions (**Figure 1C**).

**Figure 3:**
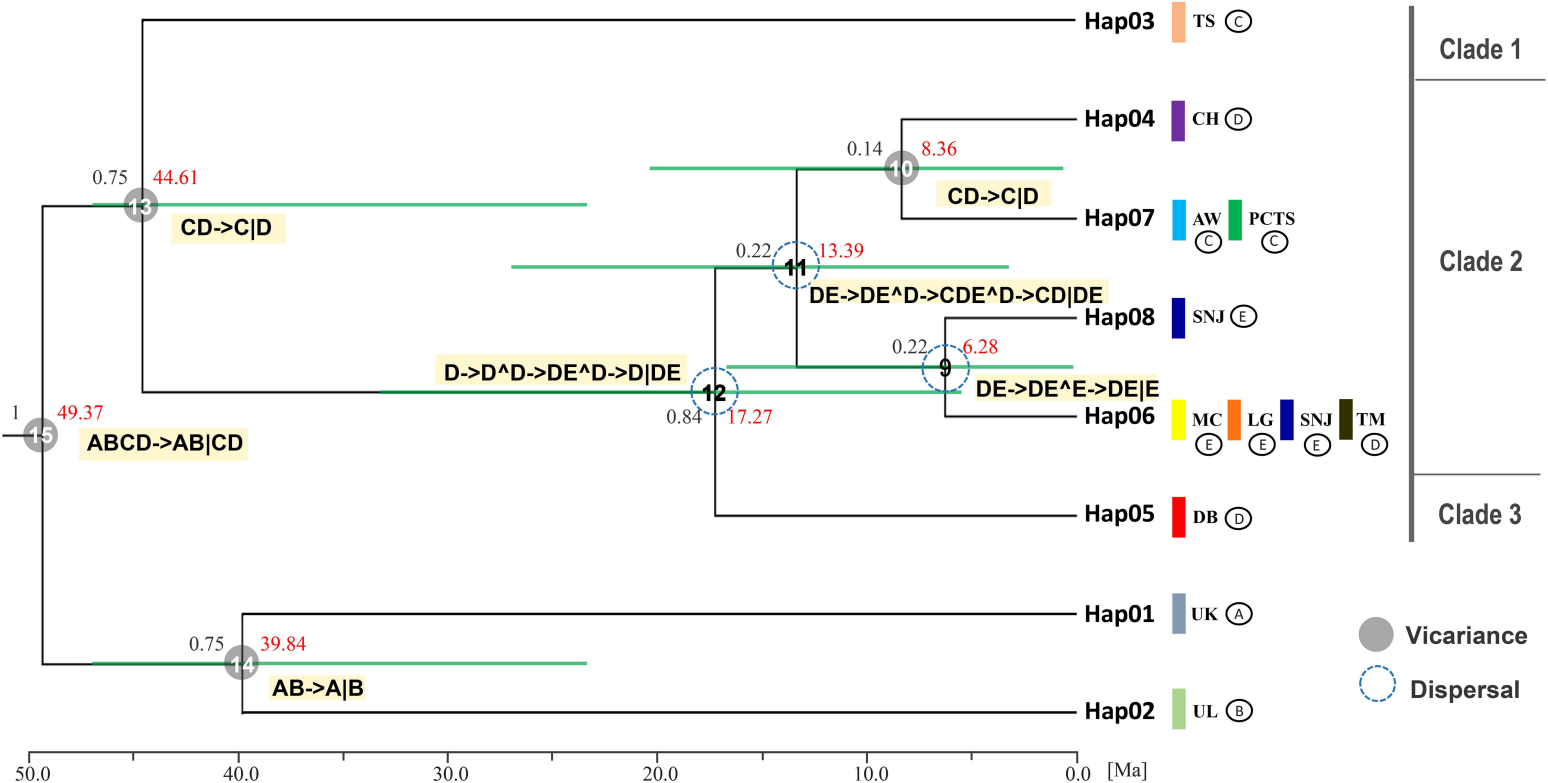
Clock-calibrated BEAST tree for chloroplast DNA haplotype of *F. hayatae*. The black numbers above the branches are posterior probabilities and node ages (Million years ago) are labeled in the node with red numbers. The horizontal green bars through these nodes indicate 95% highest posterior densities (HPD) intervals of divergence dates estimates for each clade. The vertical color bars represent different populations, while symbols **(A–E)** denote different geographic regions: **(A, B)** correspond to the outgroups, **(C)** to the TW region, **(D)** to the ZJ region, and **(E)** to the HS region. Statistical dispersal–vicariance analysis generated in RASP, the gray solid circles and blue dotted circle on nodes display the dispersal and vicariance event, the symbol (**^**) indicating dispersal and (**∣**) indicating vicariance.

Bayesian inference (BI) analysis of *F. hayatae* chloroplast DNA haplotypes revealed three monophyletic clades (**Figure 3**). Clade 1, which comprised the TS population (Hap3), was moderately supported (PP = 0.75), while the other two clades showed stronger support. In Bayesian phylogenetics, posterior probabilities of 0.95 or higher are conventionally considered strong statistical support, whereas values between 0.70 and 0.94 represent moderate support. Clade 3 consisted solely of the DB population (Hap5), emerged as the earliest diverging lineage among mainland populations. Clade 2 included both island and mainland populations but showed no clear geographic structure. Notably, the island populations PCTS and AW clustered most closely with the mainland CH population, which is geographically closest to Taiwan (**Figure 3**).

Divergence time estimates, based on a substitution rate of 1.52 × 10^-9^ s/s/y, provided further insights into the evolutionary timeline of *F. hayatae* populations (**Figure 3**). Divergence from other *Fagus* species occurred approximately 49.37 million years ago (Ma). The TS population was the first to diverge from other *F. hayatae* populations around 44.61 Ma, followed by the DB population, which separated from other mainland Chinese *F. hayatae* populations around 17.27 Ma. The PCTS and AW populations diverged from the CH population approximately 8.36 Ma (**Figure 3**). It is important to note, however, that these divergence times are based on a single chloroplast DNA intergenic region (*atp*B-*rbc*L). Such single-locus estimates can provide only an approximate timeline and are subject to considerable uncertainty, as reflected by the wide 95% HPD intervals in our analysis (**Figure 3**). These estimations should therefore be interpreted with caution.

### 3.4 Demographic history and historical biogeography inference

Tajima’s *D* and *Fu* and Li’s D* tests were conducted to evaluate the neutrality of genomic regions by comparing observed nucleotide diversity. The results indicated that values ranged from 0.23 to 3.18 for Tajima’s *D* and from 0.52 to 1.00 for Fu and Li’s D*. Notably, significantly positive Tajima’s *D* values were observed exclusively for the island populations, suggesting the influence of population bottlenecks or balancing selection in these groups. (**Supplementary Table S1**).

To assess demographic changes, a mismatch distribution analysis was performed by calculating both the sum of squared deviations (SSD) and Harpending’s raggedness index (*H*_Rag_). Most populations showed low and statistically significant values under both demographic and spatial expansion models, indicating that these *F. hayatae* populations did not experience significant expansion. Instead, factors such as bottlenecks, population structure, or other historical events likely influenced their current genetic patterns. An exception was found in the SNJ population of the HS region, where the mismatch distribution supported an expansion model (**Supplementary Table S4**).

Further insights into historical demographic changes were provided by a Bayesian skyline plot, which revealed a recent decline in effective population size in the island populations, suggesting that local genetic diversity might be at risk. In contrast, the mainland populations remained relatively constant and stable in their population sizes. Overall, when considering *F. hayatae* as a whole, the effective population size appears to have decreased over the past million years (**Supplementary Figure S1**).

**Figure 4:**
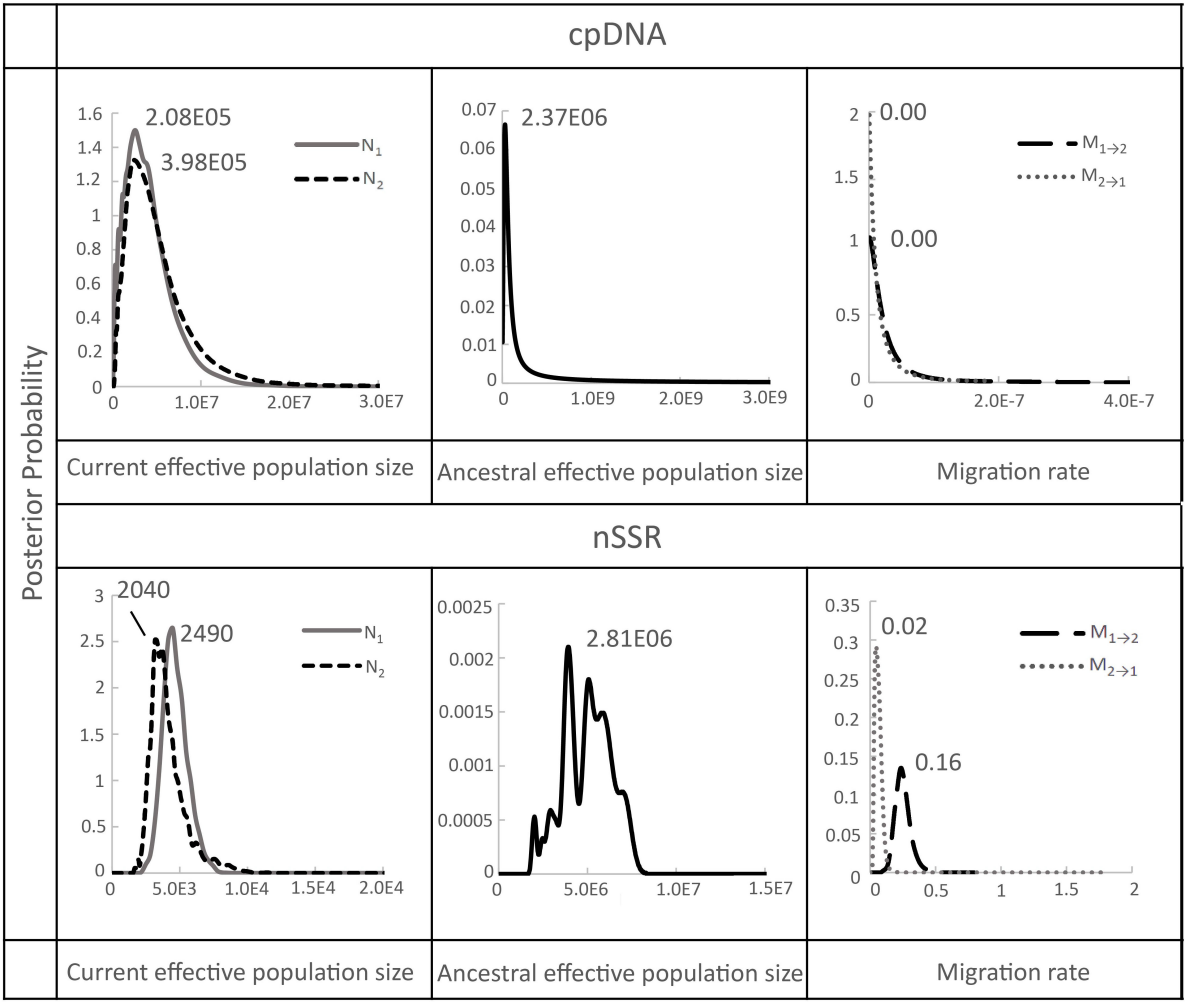
Marginal posterior probability density distribution of migration rates, and effective population sizes estimated by IMa analyses conducted between Taiwan (population 1) and China (population 2) populations of *F. hayatae*. All parameters were scaled by a mutation rate of 1.52 ± 0.06 × 10^−9^ substitutions per site per year for chloroplast noncoding regions by Yamane et al. (2006) and 8.87 × 10^-4^ (2.03 × 10^-3^-4.96 × 10^-5^) per allele per generation for microsatellite by Marriage et al. (2009).

After confirming significant genetic differentiation between island and mainland populations, we applied a coalescent-based Isolation-with-Migration (IM) model to estimate gene flow rates and effective population sizes between the island (population 1) and mainland (population 2) groups (**Supplementary Table S5**; **Figure 4**). The IMa analysis showed that the ancestral effective population sizes (N_A_) were considerably larger than the current sizes with estimates of 2.37 × 10^6^ (95% CI: 2.47 × 10^6^–2.28 × 10^6^) based on chloroplast DNA, and 2.81 × 10^6^ (95% CI: 1.23 × 10^6^–5.03 × 10^7^) based on microsatellite data. By comparison, current effective population sizes were much smaller: 2.08 × 10^5^ (95% CI: 2.17 × 10^5^–2.00 × 10^5^) for the island and 3.98 × 10^5^ (95% CI: 4.14 × 10^5^–3.83 × 10^5^) for the mainland based on chloroplast DNA, and 2,490 (95% CI: 1,090–44,500) for the island and 2,040 (95% CI: 891–36,500) for the mainland based on microsatellites (**Supplementary Table S5**; **Figure 4**). Historical gene flow estimates were generally low. Microsatellite data indicated slightly higher gene flow from Taiwan (TW) to mainland China (ZJ and HS regions) (M_1_→_2_ = 0.16, 95% CI: 0.01–0.35) than in the reverse direction (M_2_→_1_ = 0.02, 95% CI: 0.00–0.05), whereas chloroplast DNA data showed no evidence of gene flow in either direction (**Supplementary Table S5**, **Figure 4**).

Ancestral range reconstruction using RASP with chloroplast DNA data provided insights into the historical biogeography (vicariance and dispersal events) of *F. hayatae* across mainland and island regions (**Figure 3**). The results revealed several dispersal events among ancestral regions on the mainland at nodes 9, 11, and 12, with ancestors originating from the ZJ region. Additionally, two vicariance events were identified between the island and mainland, with no evidence of gene flow between them (**Figure 3**).

These findings suggest that geographic isolation has played a key role in shaping the evolutionary history and genetic divergence of *F. hayatae* between mainland and island populations.

## 4 Discussions

### 4.1 Contrasting genetic patterns between island and mainland *F. hayatae* populations - refugium theory and population dynamics

Genetic diversity analysis of *F. hayatae* revealed generally low diversity across the species, with island populations displaying higher genetic diversity but also much higher inbreeding coefficients than mainland populations. These distinct evolutionary patterns between island and mainland groups reflect their unique evolutionary histories and ecological conditions. These results support the hypothesis that Taiwan serves as a genetic refugium for *F. hayatae*. According to refugia theory, geographical isolation and climatic stability enable specific regions to become species refuges, maintaining higher genetic diversity (Hewitt, 2000). Climatic refugia are linked to areas of local or regional climate stability and biodiversity hotspots, where stable climates allow both ancient species and newly evolving lineages to survive and thrive (Harrison and Noss, 2017; Keppel et al., 2018). García et al. (2022) confirmed that stable climatic conditions and topographical complexity contribute to the accumulation of evolutionary uniqueness.

The elevated genetic diversity in Taiwanese populations aligns with refugia theory, attributed to the island’s geographic isolation, stable climate, and complex topography, which have facilitated the maintenance and accumulation of genetic variation. Previous community surveys indicated that Taiwanese *F. hayatae* populations prefer warm, humid environments with minimal seasonal variation (Shen et al., 2015), further supporting Taiwan’s role as a climatic refuge that enables long-term independent evolution and preservation of higher genetic diversity.

Our phylogenetic analysis further supports Taiwan’s role as a genetic refugium. The TS population in Taiwan represents the earliest diverging evolutionary lineage and possesses unique haplotypes, highlighting its genetic distinctiveness. These findings support Taiwan’s role as a climatic refugium and demonstrate how stable climate and topographical conditions have fostered evolutionary uniqueness (García et al., 2022). However, our analysis revealed that Taiwanese populations have higher inbreeding coefficients, primarily due to limited gene flow between isolated *F. hayatae* populations caused by long-term geographical separation. Habitat fragmentation and smaller population sizes have increased the risk of inbreeding. These factors affect genetic diversity maintenance and may reduce the populations’ long-term adaptive potential. Population dynamics analysis also revealed that current effective population sizes of Taiwanese *F. hayatae* populations have significantly decreased compared to their ancestral levels, indicating a recent genetic decline.

In contrast, mainland populations of *F. hayatae* showed lower genetic diversity compared to Taiwanese populations, possibly due to historical bottleneck effects and greater climate fluctuations in their habitat. Previous community surveys indicated that mainland Chinese *F. hayatae* populations are adapted to colder and drier climates (Shen et al., 2015), which may reflect the region’s greater climatic variability and its weaker effect on maintaining genetic diversity. However, despite their lower genetic diversity, mainland populations had lower inbreeding coefficients than Taiwanese populations. This may be because they occupy wider geographic areas with more varied environments, which likely promotes gene flow between populations and helps reduce inbreeding. Notably, our results indicated that some mainland populations, such as the TM population, showed no significant deviation from Hardy-Weinberg equilibrium, indicating stable population dynamics.

Demographic analyses confirmed that while most *F. hayatae* populations have experienced bottleneck effects or balancing selection, mainland populations exhibit stable population dynamics. Demographic analysis revealed that the SNJ population showed signs of expansion, and Bayesian skyline plots confirmed stable population sizes across mainland regions, demonstrating sustained population stability of mainland populations. However, IMa analysis revealed a significant decrease in current effective population sizes compared to ancestral populations in mainland populations. This indicates that although mainland populations maintain gene flow and show lower inbreeding levels, they have experienced reduced genetic diversity due to historical bottleneck effects and climate fluctuations.

### 4.2 The Phylogenetic pattern and population genetic structure of *F. hayatae*

Our study uncovered clear genetic differentiation between Taiwanese and mainland Chinese populations of *F. hayatae*, as evidenced by four distinct genetic clusters identified through microsatellite data and a unique genetic lineage of the Taiwanese TS population revealed by chloroplast DNA analysis. Bayesian phylogenetic analysis further delineated three well-supported monophyletic clades, with early divergence observed in the TS population (Clade 1) and the DB mainland population (Clade 3), along with a mixed cluster (Clade 2) containing both island and mainland individuals. The presence of unique haplotypes in Taiwan, particularly within the TS population, suggested that island populations had undergone independent evolutionary trajectories, likely driven by local adaptation and historical vicariance. Additionally, IMa gene flow analysis revealed extremely low bidirectional gene flow between Taiwan and mainland China, while chloroplast DNA data showed no evidence of such exchange, highlighting the strong effect of geographic isolation on the genetic structure.

The fine-scale spatial genetic structure of *F. hayatae*, characterized by increased inbreeding and distinct genetic patterns in small and isolated Taiwanese populations, is primarily shaped by gene flow patterns through pollen and seed dispersal mechanisms. Restricted pollen dispersal often leads to consanguineous mating among nearby individuals. Although wind-dispersed pollen exhibits greater dispersal distances, its distribution patterns remain stochastic and difficult to predict, while seed dispersal typically has a more pronounced impact on genetic structuring due to its localized nature (Berg and Hamrick, 1994; Angbonda et al., 2021). When both mechanisms are limited, as observed in *F. hayatae*, distinct spatial genetic patterns emerge alongside increased inbreeding and reduced diversity. In Taiwan, the dispersal of seeds and pollen in *F. hayatae* is limited. While wind serves as the primary pollination agent, the persistently high humidity and heavy rainfall in its habitat inhibit effective pollen dispersal (Chen et al., 2011), further reinforcing genetic isolation and promoting inbreeding. These environmental constraints, together with random genetic drift, accelerate divergence and reduce genetic diversity in these small, isolated populations (Ellstrand and Elam, 1993).

Integrating these findings, our results highlight that ecological constraints, historical isolation, restricted seed and pollen dispersal, and ongoing genetic drift have created strong barriers to gene flow, resulting in pronounced genetic structuring across the species’ range. The concordance between genetic patterns and ecological observations underscores the necessity to maintain or restore connectivity among fragmented populations as a core conservation priority for *F. hayatae*.

### 4.3 Evolutionary trajectories and climate-driven phylogeographical patterns of *F. hayatae*

The distinct biogeographical characteristics of China/East Asia, with its complex topography and unique east-west oriented mountain ranges, have served as crucial buffers against climatic fluctuations during the late Neogene and Quaternary periods, particularly in south-central China and adjacent regions where Tang et al. (2018) documented exceptional climatic stability compared to surrounding Asian territories (Manchester and Tiffney, 2001; López-Pujol et al., 2011; Tang et al., 2018). This long-term environmental stability, characterized by both geological and climatic constancy, has been instrumental in promoting species diversification and facilitating evolutionary processes among temperate forest floras. Similarly, Jiang et al. (2020) demonstrated that genetic differentiation patterns in *Fagus* species closely correlate with these geological and climatic stability patterns (Jiang et al., 2020). Our study on *F. hayatae* provides further evidence for this correlation.

Our phylogeographic analysis, based on the cpDNA *atp*B-*rbc*L region, reconstructs the historical dynamics of *F. hayatae* and provides a preliminary timeline of divergence events. The results suggest that *F. hayatae* diverged from other Fagus species ca. 49.37 Ma, during the middle Eocene. A significant vicariance event is estimated to have occurred ca. 44.61 Ma, separating the Taiwanese TS population lineage from other populations. We initially attributed this deep split to major climatic shifts during the Late Eocene. However, we must explicitly acknowledge the limitations inherent in using a single, non-coding chloroplast DNA marker for divergence time estimation. Such single-locus approaches can be influenced by factors like incomplete lineage sorting, potential calibration errors, or insufficient fossil constraints, which can lead to considerable uncertainty in age estimates. This uncertainty is reflected in the wide 95% highest posterior density (HPD) intervals in our analysis.

Crucially, the estimated Eocene divergence of the TS lineage presents a geological contradiction, as it significantly predates the tectonic uplift of Taiwan as a substantial landmass, which began around the mid-Miocene (~9 Ma) and intensified in the last 5 million years (Sibuet and Hsu, 2004). It is therefore unlikely that this lineage was isolated *in situ* on Taiwan since the Eocene. One plausible, though speculative, scenario is that this ancient cpDNA lineage persisted on the Asian mainland for tens of millions of years before one of its descendant populations colonized the newly formed island of Taiwan.

This discrepancy is further highlighted when comparing our results with Jiang et al. (2020) studies using 28 nuclear single/low-copy loci, estimated a considerably more recent divergence time for *F. hayatae* from its sister species (*F. crenata*) at approximately 7.8 Ma (95% HPD: 6.49–9.41 Ma). Furthermore, their analysis placed the major radiation of the East Asian, North American, and West Eurasian lineages of subgenus Fagus around the late Miocene (10.9–10.4 Ma). This timeframe aligns much better with the geological history of Taiwan’s formation and major climatic cooling events of the Neogene that drove species differentiation (Wen, 1999; Milne and Abbott, 2002). Therefore, while our cpDNA data compellingly identifies the TS population as a uniquely ancient and distinct maternal lineage, the ~44 Ma divergence estimate should be interpreted with caution and viewed as an approximate timeline requiring further validation. The multi-locus nuclear data from Jiang et al. (2022) likely provide a more realistic estimate for the species-level divergence events. Future research integrating genomic data from multiple nuclear loci will be essential to resolve this conflict between organellar and nuclear genomes and to establish a more precise and robust evolutionary timeline for *F. hayatae*.

In contrast, the estimated divergence of the AW and PCTS populations at 8.36 Ma aligns well with the geological history of Taiwan and major climatic shifts in East Asia. The island populations, AW and PCTS, became completely isolated from mainland populations during the late Miocene to early Pliocene. This period was marked by intensified global cooling, the gradual disconnection of the Bering Land Bridge, and regional species isolation due to land uplift and climate diversification in East Asia (Wen, 1999; Milne and Abbott, 2002; Ian Milne, 2006). Regional geological events and climatic shifts drove species isolation and differentiation, which ultimately shaped contemporary population structures. Notably, this period coincides with the formation of the island of Taiwan. Around 9 million years ago, the collision of the Philippine Sea Plate with the Eurasian Plate initiated the island’s gradual uplift (Sibuet and Hsu, 2004). This geological process not only elevated the terrain but also significantly altered local environmental conditions. The accelerated tectonic uplift of Taiwan further restricted gene flow among populations and led to regional species isolation. Consequently, the gradual formation of Taiwan island acted as a physical barrier that obstructed migration routes and, in conjunction with climatic changes, established both geographic and ecological obstacles between the AW and PCTS populations in Taiwan and those in China, thereby promoting species differentiation and evolution. These geological and ecological environmental changes not only explain *F. hayatae*’s distribution patterns but also reveal the profound impact of environmental changes on its population’s genetic structure and differentiation.

### 4.4 Taxonomic implications and conservation units

Our analysis revealed significant genetic divergence between island and mainland populations of *F. hayatae*, particularly the distinct genetic signature of the TS lineage, which has considerable implications for taxonomy and conservation. Revisiting the classification controversy mentioned earlier with our new genetic evidence and recent literature provides valuable insights. Li et al. (2023) integrated molecular and morphological data to synonymize the previously ambiguous *F. chienii* with *F. hayatae*, resolving a longstanding taxonomic question regarding mainland Chinese populations. This taxonomic consolidation aligns with our treatment of all mainland samples as members of a single, genetically structured *F. hayatae* species.

Nevertheless, our genetic findings clearly demonstrate that this taxonomic unification should not diminish the significant evolutionary distinctiveness of Taiwanese populations. The pronounced divergence of the TS population’s chloroplast haplotype, the clear genetic differentiation detected by both SAMOVA and STRUCTURE analyses, and the virtually nonexistent gene flow between Taiwan and mainland China collectively indicate that Taiwanese populations have followed a separate evolutionary pathway.

These findings raise the question of whether Taiwanese populations warrant classification as a separate subspecies or species. While formal taxonomic revision exceeds the scope of our study, our data strongly support designating Taiwanese populations, especially the TS population, as an Evolutionarily Significant Unit (ESU). This designation applies to population segments that are reproductively isolated and represent critical components of a species’ evolutionary heritage. The unique genetic composition, significant divergence, and prolonged isolation of Taiwanese lineages precisely fulfill these criteria. This genetic distinctiveness, combined with elevated inbreeding coefficients in Taiwanese populations, indicates they are following an independent evolutionary trajectory.

From a conservation perspective, this distinction is crucial. Managing *F. hayatae* as a single conservation unit across its entire distribution would be inappropriate and could result in losing valuable genetic diversity. We recommend implementing separate conservation strategies for mainland and Taiwanese populations. Additionally, the TS population, representing the most ancient and divergent lineage identified in our study, should receive priority for *in-situ* conservation efforts to safeguard its unique and irreplaceable genetic diversity.

## 5 Conclusion

This study tested three key hypotheses regarding *F. hayatae* phylogeographic. Our findings strongly supported the first hypothesis, demonstrating significant genetic differentiation between Taiwanese and mainland Chinese populations driven by geographic isolation and historical climatic events. The second hypothesis was also confirmed, as habitat fragmentation has resulted in high inbreeding coefficients in Taiwan. Consistent with our third hypothesis, phylogeographic reconstructions revealed deep vicariance events and limited gene flow shaping distinct evolutionary trajectories. *F. hayatae*’s current population structure results from both historical and contemporary factors. Geographic isolation, confirmed through IMa analysis and chloroplast DNA, has been a primary driver of differentiation. Taiwan’s topography and stable climate served as a crucial genetic refugium, preserving higher genetic diversity compared to mainland populations. Taiwanese populations harbor exclusive haplotypes with high inbreeding coefficients, while mainland populations maintain more stable dynamics with reduced inbreeding due to greater connectivity. These genetic distinctions, particularly the ancient lineage of the Taiwanese TS population, have significant conservation implications. Our data support designating Taiwanese populations as a distinct Evolutionarily Significant Unit (ESU) warranting separate conservation management. In conclusion, *F. hayatae*’s biogeographic distribution has been shaped by historical barriers, limited gene flow, and differing regional dynamics, highlighting Taiwan’s importance as a center of evolutionary novelty for this relict species.

## Data Availability

The datasets presented in this study can be found in online repositories. The names of the repository/repositories and accession number(s) can be found in the article/**Supplementary Material**.
