# Supplementary material for: Unraveling historical genetic divergence and gene flow patterns between island (Taiwan) and mainland (China) of Fagus hayatae
Source: Front Plant Sci. 2025 Jul 29;16:1628728. doi: 10.3389/fpls.2025.1628728 (PMC12339571; doi:10.3389/fpls.2025.1628728)
Supplement: Supplementary file 1 [file DataSheet1.docx]

Supplementary Material

Supplementary Table S1. Nucleotide diversity parameters estimated from chloroplast DNA data of *F. hayatae.*

| **Region/Population** | *h* | *S* | *Hs* | *H_T_* | *Hr* | *Hp* | *Hd* | π (×10^-3^) | *θw* (×10^-3^) | Fu & Li’s D* | Tajima’s D | *G_ST_* | *N_ST_* |
| --- | --- | --- | --- | --- | --- | --- | --- | --- | --- | --- | --- | --- | --- |
| **TW region** | 2 | 5 | 0.89 | 0.84 | 1.55 | 1.55 | 0.46 | 2.48 | 0.95 | 1.00 | 3.18** | 1.00 | 1.00 |
| TS | 1 | 0 | 0.90 | 0.85 | 1.00 | 0.50 | 0.00 | 0.00 | 0.00 | - | - | - | - |
| PCTS | 1 | 0 | 0.86 | 0.75 | 1.00 | 0.00 | 0.00 | 0.00 | 0.00 | - | - | - | - |
| AW | 1 | 0 | 0.86 | 0.75 | 1.00 | 0.00 | 0.00 | 0.00 | 0.00 | - | - | - | - |
| **ZJ region** | 3 | 3 | 0.89 | 0.79 | 1.32 | 1.32 | 0.49 | 1.37 | 0.79 | 0.93 | 1.67 | 1.00 | 1.00 |
| CH | 1 | 0 | 0.88 | 0.81 | 1.00 | 0.13 | 0.00 | 0.00 | 0.00 | - | - | - | - |
| DB | 1 | 0 | 0.90 | 0.72 | 1.00 | 0.25 | 0.00 | 0.00 | 0.00 | - | - | - | - |
| TM | 1 | 0 | 0.86 | 0.77 | 1.00 | 0.00 | 0.00 | 0.00 | 0.00 | - | - | - | - |
| **HS region** | 2 | 1 | 0.87 | 0.78 | 1.00 | 1.00 | 0.25 | 0.26 | 0.22 | 0.52 | 0.23 | 0.54 | 0.60 |
| SNJ | 2 | 1 | 0.88 | 0.80 | 1.00 | 0.00 | 0.50 | 0.54 | 0.32 | 0.69 | 1.31 | - | - |
| LG | 1 | 0 | 0.86 | 0.77 | 1.00 | 0.00 | 0.00 | 0.00 | 0.00 | - | - | - | - |
| MC | 1 | 0 | 0.86 | 0.77 | 1.00 | 0.00 | 0.00 | 0.00 | 0.00 | - | - | - | - |
| **Overall** | 6 | 9 | 0.88 | 0.84 | 1.78 | 1.78 | 0.74 | 2.89 | 1.57 | 1.25 | 1.88 | 0.96 | 0.97 |

*h*: number of haplotypes, *S*: number of polymorphic sites, *H_S_*: genetic diversity within populations, *H_T_*: total genetic diversity, *Hr*: haplotype richness, *Hp*: private haplotype richness, *Hd*: haplotype diversity, *π*: nucleotide diversity, *θw*: nucleotide polymorphism, *G_ST_, N_ST_*: genetic differentiation for and phylogeographic signal test, ** *p*<0.01.

Supplementary Table S2. Analyses of molecular variance (AMOVA) of chloroplast DNA and microsatellite data were conducted to assess genetic differentiation. The groups defined by distinct geographic groups [*K*_geo_=2 (Taiwan & China), *K*_geo_=3 (TW & HS & ZJ region), *K*_geo_=9 (9 populations)] and genetic groups [*K*_S_ (cpDNA)=2 (TS & PCTS, AW, CH, DB, TM, SNJ, MC, LG), *K*_S_ (SSR)=3 (PCTS, TS & AW & CH, DB, TM, SNJ, MC, LG), *K*_SA_ (SSR)=4 (PCTS & AW & TS & CH, DB, TM, SNJ, MC, LG].

| Clusters | Source of variation | *d.f.* | Sum of squares | Variance components | Percentage of variation | Fixation Indices |
| --- | --- | --- | --- | --- | --- | --- |
| chloroplast DNA |  | | | | | |
| *K*_geo_=2 | Among groups | 1 | 119.14 | 0.43 | 23.24 | Ф*_CT_*=0.23 |
|  | Among populations within groups | 7 | 228.74 | 1.42 | 75.97 | Ф*_SC_*=0.99* |
|  | Within populations | 253 | 3.75 | 0.01 | 0.79 | Ф*_ST_*=0.99* |
| *K*_geo_=3 | Among groups | 2 | 141.91 | 0.31 | 17.22 | Ф*_CT_*=0.17 |
|  | Among populations within groups | 6 | 205.97 | 1.47 | 81.95 | Ф*_SC_*=0.99* |
|  | Within populations | 253 | 3.75 | 0.01 | 0.83 | Ф*_ST_*=0.99* |
| *K*_geo_=9 | Among populations | 8 | 347 | 1.69 | 99.13 | Ф*_ST_*=0.99* |
|  | Within populations | 253 | 3.75 | 0.01 | 0.87 |  |
| *K*_S_=2 | Among groups | 1 | 258.69 | 1.69 | 71.31 | Ф*_CT_*=0.71* |
|  | Among populations within groups | 7 | 89.18 | 0.67 | 28.06 | Ф*_SC_*=0.98* |
|  | Within populations | 253 | 3.75 | 0.01 | 0.62 | Ф*_ST_*=0.99* |
| microsatellite |  | | | | | |
| *K*_geo_=2 | Among groups | 1 | 174.80 | 0.73 | 15.09 | Ф*_CT_*=0.15* |
|  | Among populations within groups | 7 | 217.50 | 0.76 | 15.88 | Ф*_SC_*=0.19* |
|  | Within populations | 175 | 1175.71 | 3.32 | 69.04 | Ф*_ST_*=0.30* |
| *K*_geo_=3 | Among groups | 2 | 243.94 | 0.85 | 17.92 | Ф*_CT_*=0.18* |
|  | Among populations within groups | 6 | 148.37 | 0.59 | 12.30 | Ф*_SC_*=0.15* |
|  | Within populations | 175 | 1175.71 | 3.32 | 69.78 | Ф*_ST_*=0.30* |
| *K*_geo_=9 | Among populations | 8 | 392.30 | 1.19 | 26.38 | Ф*_ST_*=0.26* |
|  | Within populations | 175 | 1175.71 | 3.32 | 73.62 |  |
| *K*_S_=3 | Among groups | 2 | 205.87 | 0.36 | 7.81 | Ф*_CT_*=0.08* |
|  | Among populations within groups | 6 | 186.43 | 0.92 | 20.02 | Ф*_SC_*=0.22* |
|  | Within populations | 175 | 1175.71 | 3.32 | 72.17 | Ф*_ST_*=0.28* |
| *K*_SA_=4 | Among groups | 3 | 247.45 | 0.289 | 6.32 | Ф*_CT_*=0.06* |
|  | Among populations within groups | 5 | 144.85 | 0.96 | 21.00 | Ф*_SC_*=0.22* |
|  | Within populations | 175 | 1175.71 | 3.32 | 72.68 | Ф*_ST_*=0.27* |

*K*_S_: Genetic groups established by STRUCTURE analysis.

*K*_SA_: Genetic groups established by SAMOVA analysis.

*: *p*<0.05.

Supplementary Table S3. STRUCTURE and SAMOVA results for testing genetic subdivision between populations to infer the number of clusters *K* in *F. hayatae* based on chloroplast DNA and microsatellite data.

|  | chloroplast DNA | | | | |  | microsatellite | | | | |
| --- | --- | --- | --- | --- | --- | --- | --- | --- | --- | --- | --- |
|  | Mean LnP(K) | *ΔK* | F*_SC_*(P) | F*_ST_* (P) | F*_CT_*(P) |  | Mean LnP(K) | *ΔK* | F*_SC_* (P) | F*_ST_*(P) | F*_CT_* (P) |
| 1 | -1032.20 | - | - | - | - |  | -9572.80 | - | - | - | - |
| 2 | -310.90 | **3818.27** | 0.19(0.00) | 0.31(0.00) | 0.15(0.01) |  | -7941.98 | 58.16 | 0.18(0.00) | 0.58(0.00) | 0.49(0.01) |
| 3 | -272.63 | 1.87 | 0.15(0.00) | 0.30(0.00) | 0.18(0.00) |  | -7041.32 | **1882.04** | 0.09(0.00) | 0.56(0.00) | 0.52(0.01) |
| 4 | -137.50 | 189.51 | 0.13(0.00) | 0.30(0.00) | 0.20(0.00) |  | -6622.93 | 0.48 | -0.03(0.00) | 0.53(0.00) | **0.55(0.01)** |
| 5 | -222.72 | 2.31 | 0.10(0.00) | 0.29(0.00) | 0.22(0.00) |  | -6200.53 | 36.92 | -0.03(0.00) | 0.53(0.00) | 0.54(0.01) |
| 6 | -214.77 | 4.47 | 0.09(0.00) | 0.29(0.00) | 0.22(0.00) |  | -6021.15 | 0.26 | -0.02(0.00) | 0.51(0.00) | 0.53(0.01) |
| 7 | -278.73 | 0.06 | 0.07(0.00) | 0.29(0.00) | 0.23(0.00) |  | -5825.95 | 0.22 | -0.02(0.00) | 0.50(0.00) | 0.51(0.01) |
| 8 | -347.00 | 0.27 | - | - | - |  | -5610.30 | 143.46 | -0.10(0.00) | 0.48(0.00) | 0.52(0.03) |
| 9 | -386.88 | - | - | - | - |  | -5500.07 | - | - | - | - |

*F_SC_*, differentiation between populations within groups; *F_ST_*, differentiation between population among groups; and *F_CT_*, differentiation between groups. Significance level is shown in parentheses. Values in bold type indicate the high proportion of membership for each population and we used in this study.

Supplementary Table S4. Summary of mismatch distribution analysis (parameters of demographic and spatial expansion) for 9 populations and distinct groups of *F. hayatae* based on chloroplast DNA data. The groups are defined in below: Q_All_ (overall populations), Q_TW_ (TS, PCTS, AW), Q_HS_ (SNJ, LG, MC), Q_ZJ_ (CH, DB, TM), Q_PL_ (PCTS, AW, CH, DB, TM, SNJ, MC, LG).

|  | Demographic expansion | |  | Spatial expansion | |
| --- | --- | --- | --- | --- | --- |
| Groups | SSD (*p*-value) | *H*_Rag_ (*p*-value) |  | SSD (*p*-value) | *H*_Rag_ (*p*-value) |
| TS | 0.000 (0.000) | 0.000 (0.000) |  | 0.000 (0.000) | 0.000 (0.000) |
| PCTS | 0.000 (0.000) | 0.000 (0.000) |  | 0.000 (0.000) | 0.000 (0.000) |
| AW | 0.000 (0.000) | 0.000 (0.000) |  | 0.000 (0.000) | 0.000 (0.000) |
| CH | 0.000 (0.000) | 0.000 (0.000) |  | 0.000 (0.000) | 0.000 (0.000) |
| DB | 0.000 (0.000) | 0.000 (0.000) |  | 0.000 (0.000) | 0.000 (0.000) |
| TM | 0.000 (0.000) | 0.000 (0.000) |  | 0.000 (0.000) | 0.000 (0.000) |
| SNJ | **0.022 (0.100)** | **0.250 (0.100)** |  | 0.022 (0.040) | **0.250 (0.120)** |
| LG | 0.000 (0.000) | 0.000 (0.000) |  | 0.000 (0.000) | 0.000 (0.000) |
| MC | 0.000 (0.000) | 0.000 (0.000) |  | 0.000 (0.000) | 0.000 (0.000) |
| Q_All_ | 0.002 (0.016) | 0.028 (0.004) |  | 0.002 (0.009) | 0.028 (0.013) |
| Q_TW_ | 0.000 (0.000) | 0.000 (0.000) |  | 0.000 (0.000) | 0.000 (0.000) |
| Q_HS_ | 0.007 (0.040) | 0.083 (0.040) |  | 0.007 (0.020) | 0.083 (0.040) |
| Q_ZJ_ | 0.000 (0.000) | 0.000 (0.000) |  | 0.000 (0.000) | 0.000 (0.000) |
| Q_PL_ | 0.003 (0.028) | 0.031 (0.013) |  | 0.003 (0.010) | 0.031 (0.010) |

The sum of squared deviation (SSD) and raggedness index (*H*_Rag_) are for testing the sudden expansion model. The bolds indicate *p*>0.05.

Supplementary Table S5. The highest posterior parameter estimate (HiPt) and lower to upper bounds of 95%HPD intervals (HPD95Lo - HPD95Hi) of demographic parameters of *F. hayatae* from IMa analysis. The effective population size (N) of population 1 (N_1_), population 2 (N_2_), ancestral population (N_A_), divergence time (T), and migration rate from population 2 into population 1 (M_1→2_) and from population 1 into population 2 (M_2→1_) are estimated. The parameter are scaled by the mutation rate of 1.52 ± 0.06 × 10^−9^ substitutions per site per year for chloroplast DNA by Yamane et al. (2006) and 8.87 × 10^-4^ (2.03 × 10^-3^- 4.96 × 10^-5^) per allele per generation for microsatellite by Marriage et al. (2009).

|  | *θ_1_* | *θ_2_* | *θ_A_* | *m_1→2_* | *m_2→1_* | *t* | N_1_ | N_2_ | N_A_ | M_1→2_ | M_2→1_ | T (× years) |
| --- | --- | --- | --- | --- | --- | --- | --- | --- | --- | --- | --- | --- |
| chloroplast DNA | | | | | | | **μ=1.52 × 10^−9^ (1.46 × 10^−9^-1.58 × 10^−9^)** | | | | | |
| HiPt | 0.32 | 0.30 | 3.74 | 0.02 | 0.00 | 0.19 | 2.60E06  (2.70E06-2.50E06) | 2.48E06 (3.21E07-2.39E06) | 3.08E07  (3.21E07-2.96E07) | 0.00  (0.00- 0.00) | 0.00  (0.00- 0.00) | 1.22E08  (1.27E08-1.17E08) |
| HPD95  Lo | 0.03 | 0.05 | 0.29 | 0.01 | 0.00 | 0.04 | 2.08E05  (2.17E05-2.00E05) | 3.98E05  (4.14E05-3.83E05) | 2.37E06  (2.47E06-2.28E06) | 0.00  (0.00- 0.00) | 0.00  (0.00- 0.00) | 2.30E07  (2.40E07-2.22E07) |
| HPD95  Hi | 0.94 | 1.23 | 230.11 | 2.65 | 2.01 | 1.14 | 7.75E06  (8.07E06-7.45E06) | 1.01E07  (1.05E07-9.72E06) | 1.89E09  (1.97E09-1.82E09) | 0.00  (0.00- 0.00) | 0.00  (0.00- 0.00) | 7.47E08  (7.77E08-7.18E08) |
| microsatellite | | | | | | | **μ=8.87 × 10^-4^ (2.03 × 10^-3^- 4.96 × 10^-5^)** | | | | | |
| HiPt | 0.79 | 0.56 | 696.01 | 13.33 | 2.55 | 0.02 | 4480  (1960-80100) | 3180  (1390-56800) | 3.92E06  (1.71E06-7.02E07) | 0.24  (0.54-0.01) | 0.05  (0.10-0.00) | 552  (241-9880) |
| HPD95  Lo | 0.44 | 0.36 | 498.83 | 8.73 | 1.25 | 0.01 | 2490  (1090-44500) | 2040  (891-36500) | 2.81E06  (1.23E06-5.03E07) | 0.16  (0.35-0.01) | 0.02  (0.05-0.00) | 237  (103-4230) |
| HPD95  Hi | 1.32 | 1.09 | 1308.15 | 19.33 | 5.65 | 0.07 | 7470  (3260-133000) | 6120  (2670-109000) | 7.37E06  (3.22E06-1.32E08) | 0.34  (0.79-0.02) | 0.1  (0.23-0.01) | 1500  (6550-26800) |


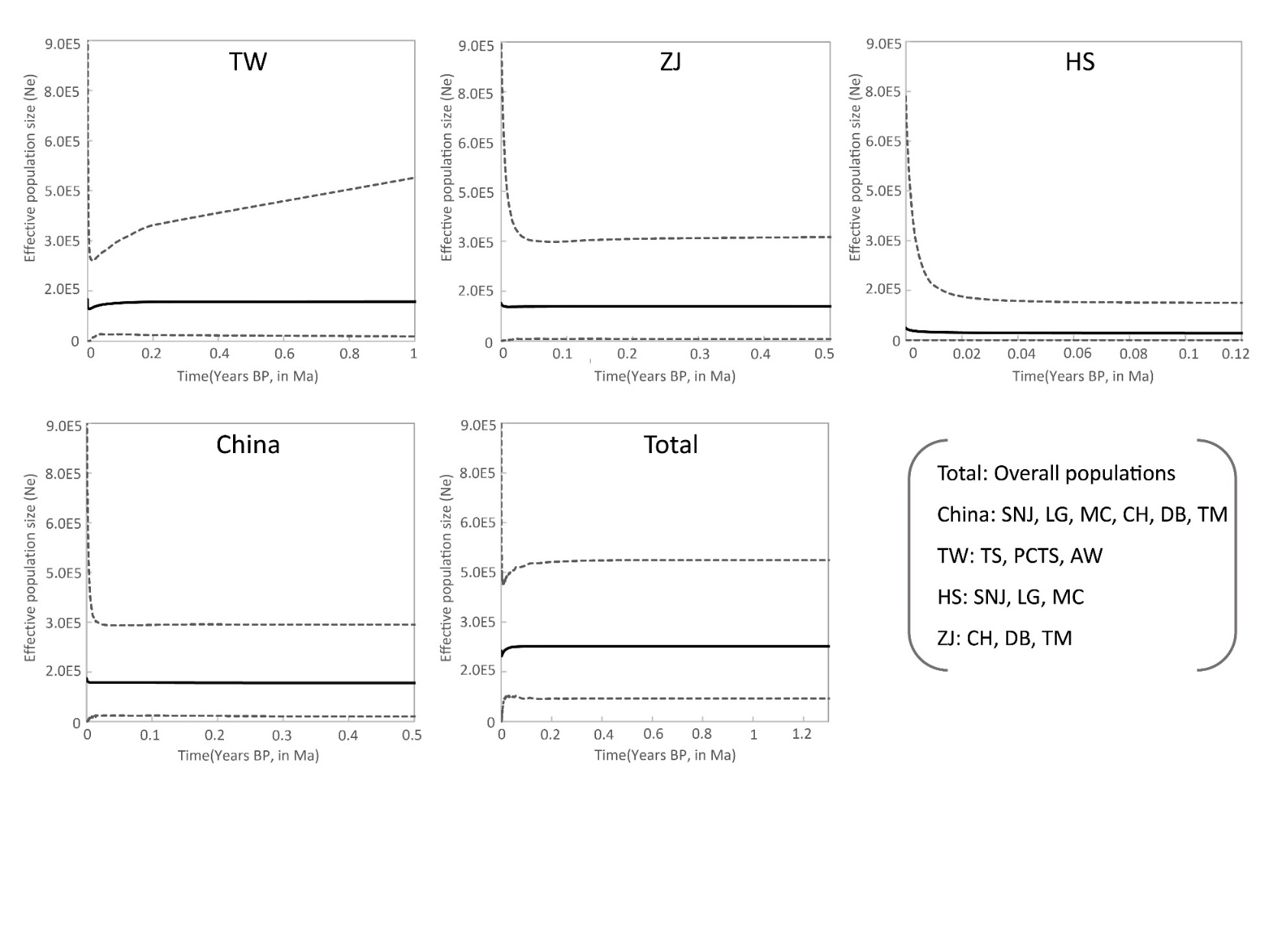
Supplementary Figure S1. Bayesian skyline plots depicting historical demographic changes for major geographical regions of *F. hayatae*. The effective population size is shown as a function throughout time (years before present, in Ma). The solid line represents the median estimate of population size; the dotted lines show the confidence intervals at 95% highest posterior density (HPD) limits of the effective population size.
